# Complete chloroplast genome of *Actaea heracleifolia* (Kom.) J. Compton

**DOI:** 10.1080/23802359.2018.1502636

**Published:** 2018-08-23

**Authors:** Inkyu Park, Sungyu Yang, Wook Jin Kim, Pureum Noh, Hyun Oh Lee, Byeong Cheol Moon

**Affiliations:** a Herbal Medicine Research Division, Korea Institute of Oriental Medicine, Daejeon, Republic of Korea;; b Phyzen Genomics Institute, Seongnam, Republic of Korea

**Keywords:** *Actaea heracleifolia*, herbal medicine, chloroplast genome, Ranunculaceae

## Abstract

Dried rhizomes of *Actaea heracleifolia*, used as a traditional Korean herbal medicine, are frequently adulterated with other plant species. For accurate species identification, we sequenced the complete chloroplast genome of *A. heracleifolia* using Illumina MiSeq. *A. heracleifolia* harbours a 159,578 bp chloroplast genome comprising a large single-copy region (88,770 bp), small single-copy region (18,070 bp) and two inverted repeat (IR) regions (IRa and IRb; each 26,519 bp). The chloroplast genome contains 112 unique genes, including 78 protein-coding genes, 4 ribosomal RNA genes, and 30 transfer RNA genes. Phylogenetic analysis revealed that *A. heracleifolia* was closely related to *Gymnaconitum gymnandrum*.

*Actaea heracleifolia* (Kom.) J. Compton belongs to the family Ranunculaceae, which is widely distributed across the world (Compton et al. 1998). Dried rhizomes of *A. heracleifolia*, Cimicifugae Rhizoma, are used as a traditional herbal medicine in Korea to treat wind-heat headache, toothache, aphtha, sore throat, measles, and spot poison (KIOM 2018). Although dried rhizomes of *A. heracleifolia* are a valuable herbal medicine, they are frequently mixed and indiscriminately misused with other similar plants in the Korean herbal market (Moon et al. 2017). Therefore, to accurately identify and discriminate *A. heracleifolia* from other similar plants, we sequenced its complete chloroplast genome.

Fresh leaves of *A. heracleifolia* were collected from its native habitat in Korea (37° 35′ 17.6″ N and 128° 35′ 5.3″ E) and registered at the Korea Institute of Oriental Medicine (KIOM) in the Korean Herbarium of Standard Herbal Resources (Index Herbariorum code KIOM) under the voucher number KIOM201401011038. Genomic DNA was extracted from the leaf samples using DNeasy Plant Maxi Kit (QIAGEN, Valencia, CA). An Illumina paired-end library was construed and sequenced using MiSeq platform (Illumina Inc., San Diego, CA). The complete chloroplast genome of *A. heracleifolia* was deposited in the GenBank database of the National Center for Biotechnology Information under the accession number MH539824.

Illumina sequencing of *A. heracleifolia* generated approximately 2.7 Gb of high-quality paired-end reads. The complete chloroplast genome of *A. heracleifolia* was 159,578 bp in length and showed a typical quadripartite structure, consisting of an 88,770 bp large single-copy (LSC) region, a 7022 bp small single-copy (SSC) region and 14,200 bp each of two inverted repeat (IR) regions (IRa and IRb). The GC content of the complete chloroplast genome was 38.1%, with the IR regions showing a higher GC content (43.1%) than the LSC (36.2%) and SSC (32.4%) regions. These data suggest that the chloroplast genome of *A. heracleifolia* is AT-rich. The GC content of *A. heracleifolia* chloroplast genome was 37.9%, with IR regions showing a higher GC content (43.2%) than the LSC (36%) and SSC (29.6%) regions. The chloroplast genome of *A. heracleifolia* contained 112 unique genes, including 78 protein-coding genes, 30 transfer RNA genes, and 4 ribosomal RNA genes. The coding sequence of *rpl32* carried a partial deletion and was, therefore, labelled as a pseudogene.

To identify the phylogenetic relationship of *A. heracleifolia* with other plant species, 74 protein-coding gene sequences of *A. heracleifolia* and 14 other species were aligned, and a phylogenetic tree was constructed using the maximum likelihood (ML) method (Figure 1). The tree revealed nine nodes, each with 100% bootstrap values, thus supporting a strong phylogenetic relationship within the family Ranunculaceae. *A. heracleifolia* formed a monophyletic group with other species in the family Ranunculaceae, including *Gymnaconitum gymnandrum*, *Aconitum coreanum*, and *A. pseudolaeve* (Figure 1).

**Figure 1.**
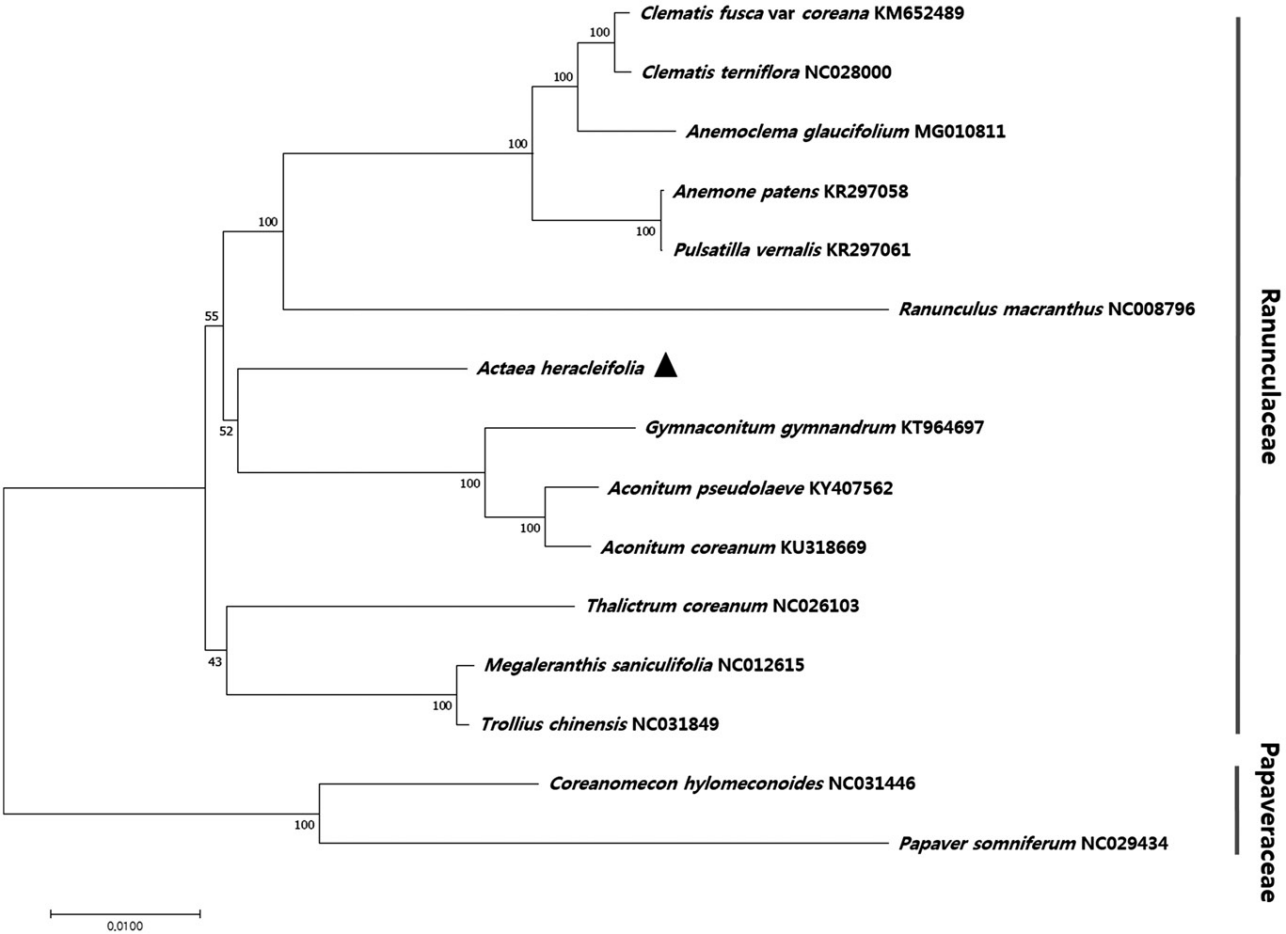
Phylogenetic analysis of the relationship between *Actaea heracleifolia* and 14 other plant species, including two out-groups. The phylogenetic tree was constructed using the maximum likelihood (ML) method. Sequences of 74 chloroplast protein-coding genes were aligned using MAFFT (Katoh et al. 2002) and subjected to phylogenetic analysis in MEGA 6.0 (Tamura et al. 2013). Bootstrap support values from 1000 replicates are indicated at the nodes.
